# Surgical strategy for management of L1-L2 disc, based on unique differences with other lumbar levels

**Published:** 2012-11

**Authors:** Kaveh Ebrahimzadeh, Guive Sharifi, Misagh Shafizad

**Affiliations:** ^a^Department of Neurosurgery , Loghman Hakim Hospital, Shaheed Beheshti University of Medical Sciences, Tehran, Iran.

## Abstract

**Background:**

Because of the scarceness and absence of hectic painful radiculopathic feature of L1-L2 disc, surgeons don’t usually pay enough clinical attention to them. Their main manifestation is severe axial pain or myeloradiculopathy failure attributed to conus or high cauda equine syndrome.

**Methods:**

During 2006 to 2011, a total of 11 patients with L1 L2 dysfunctions underwent a surgery. Their mean age was 56 years old and sex ratio of 6 male to 7 female. 4 of them had florid neurological deficit as paraparesis and sphincter dysfunction. In other patients, radiculopathies and axial pain were dominant clinical manifestations.

Considering the conus position and the extent of canal compromise and disc consistency (presence of calcification) either anterior or posterior surgical approach followed by segmental fusions was used.

**Results:**

3 patients underwent thoracoabdominal and fusion surgery, while ten underwent a posterior transfacet pedicle sparing surgery combined with transforaminal lumbar interbody fusion (TLIF) surgery. In all patients, satisfactory outcomes were achieved; all radiculopathy and signs of conus dysfunction were improved except one case that showed sphincter dysfunction persistent post operation. Axial pain was resolved and in follow-up assessments no kyphosis or axial pain were observed.

**Conclusions:**

Due to the biomechanical traits of L1-L2 disc and its adjacency to thoracolumbar region as well as high degree of osteodiscal degeneration on its manifestation we propose TLIF and Posterior spinal fusion (PSF) surgical approaches after canal decompression to alleviate axial pain and also prevent later kyphosis. Furthermore, because of nearby conus and the risk of thecal sac retraction, the same strategy as thoracic disc was performed in this region considering reverence for conus if good results are to be achieved.

**Keywords:**

Surgical strategy, L1-L2 disc, Thoracoabdominal, Transforaminal lumbar interbody fusion


					Volume 4, Suppl. 1 Nov 2012
Publisher: Kermanshah University of Medical Sciences
URL: http://www.jivresearch.org
Copyright:
Congress of Iranian Neurosurgeons, 14-16 November 2012, Kermanshah, Iran
Paper No. 74
Surgical strategy for management of L1-L2 disc, based on
unique differences with other lumbar levels
Kaveh Ebrahimzadeh a
, Guive Sharifi a
, Misagh Shafizad a,*
a
Department of Neurosurgery, Loghman Hakim Hospital, Shaheed Beheshti University of Medical Sciences, Tehran, Iran.
Abstract:
Background: Because of the scarceness and absence of hectic painful radiculopathic feature of L1-L2 disc, surgeons
don't usually pay enough clinical attention to them. Their main manifestation is severe axial pain or
myeloradiculopathy failure attributed to conus or high cauda equine syndrome.
Methods: During 2006 to 2011, a total of 11 patients with L1 L2 dysfunctions underwent a surgery. Their mean
age was 56 years old and sex ratio of 6 male to 7 female. 4 of them had florid neurological deficit as paraparesis
and sphincter dysfunction. In other patients, radiculopathies and axial pain were dominant clinical manifestations.
Considering the conus position and the extent of canal compromise and disc consistency (presence of calcification)
either anterior or posterior surgical approach followed by segmental fusions was used.
Results: 3 patients underwent thoracoabdominal and fusion surgery, while ten underwent a posterior transfacet
pedicle sparing surgery combined with transforaminal lumbar interbody fusion (TLIF) surgery. In all patients,
satisfactory outcomes were achieved; all radiculopathy and signs of conus dysfunction were improved except one
case that showed sphincter dysfunction persistent post operation. Axial pain was resolved and in follow-up
assessments no kyphosis or axial pain were observed.
Conclustions: Due to the biomechanical traits of L1-L2 disc and its adjacency to thoracolumbar region as well as
high degree of osteodiscal degeneration on its manifestation we propose TLIF and Posterior spinal fusion (PSF)
surgical approaches after canal decompression to alleviate axial pain and also prevent later kyphosis. Furthermore,
because of nearby conus and the risk of thecal sac retraction, the same strategy as thoracic disc was performed in
this region considering reverence for conus if good results are to be achieved.
Keywords:
Surgical strategy, L1-L2 disc, Thoracoabdominal, Transforaminal lumbar interbody fusion
* Corresponding Author at:
Misagh Shafizad: Neurosurgery Department, Loghman Hakim Hospital, Shaheed Beheshti University of Medical Sciences, Tehran, Iran.
Tel: 09111540432, Email: mishafizad@gmail.com, (Shafizad M.).

				

